# Genetic characterization of some Turkish sheep breeds based on the sequencing of the *Ovar-DRB1* gene in the major histocompatibility complex (MHC) gene region

**DOI:** 10.5194/aab-61-475-2018

**Published:** 2018-12-06

**Authors:** Fulya Özdil, Fatma İlhan, Raziye Işık

**Affiliations:** 1 Dept. of Agricultural Biotechnology, Namık Kemal University, Tekirdağ, Turkey; 2 Dept. of Animal Science, Selçuk University, Konya, Turkey

## Abstract

In this research, *Ovar-DRB1* gene in the major histocompatibility complex (MHC) gene region was surveyed by DNA sequencing in some of the native sheep breeds that are reared in Turkey. A total of 80 samples were collected from eight different Turkish native sheep breeds, and these samples were used for DNA sequencing.

The exon 2 region of *Ovar-DRB1 *in the MHC gene region was polymerase chain reaction (PCR) amplified and sequenced. A total of 25 new alleles were revealed in the *Ovar-DRB1* gene in Turkish native sheep breeds with 24 variable sites; only 13 sites were parsimony informative. The average pairwise genetic distance was 0.029 % for the *Ovar-DRB1* gene exon 2 region. The sequence variations at eight different positions (7026, 7036, 7040, 7053, 7059, 7069, 7131 and 7214) are found in all of the studied samples. G → C transversion at position 7081 is only seen in Akkaraman sheep breed, whereas T → C transition at position 7097 is only seen in one sample from the Akkaraman breed. Overall, two main groups were detected among the 25 alleles from Turkish native sheep breeds. All Daǧliç and Kivircik alleles and one allele from Karayaka, Malya and Sakiz are grouped together while all the other breeds are grouped in the other branch.

## 1 Introduction

Diseases are the most important factor which decrease the productivity in animal husbandry. Animal husbandry farms lose money due to diseases and deaths. Therefore, diseases must be reduced or eliminated. Cure is expensive and takes a long time after the animals are sick. Therefore, if the immune system of the animals can be developed, this may avoid or reduce the diseases.

**Table 1:** Variable sites of the *Ovar-DRB1* gene exon 2 region in Turkish native sheep breeds.

Allele/reference	GenBank	7026	7036	7040	7053	7059	7069	7081	7083	7095	7097	7131	7133	7146	7149	7174	7175	7180	7203	7204	7214	7217	7222	7227	7261
	accession																								
	numbers																								
Ballingall	AM884914	T	C	A	G	C	G	G	A	A	T	A	G	A	A	G	A	A	C	T	A	C	G	G	A
et al. (2008)${}^{*}$																									
AK1	MH686535	C	T	C	A	T	C	.	T	.	.	G	.	.	.	.	.	.	.	.	G	.	.	T	T
AK2	MH686536	C	T	C	A	T	C	.	T	.	.	G	.	T	G	A	.	G	G	A	G	.	A	T	T
AK3	MH686537	C	T	C	A	T	C	C	T	.	C	G	.	.	.	A	.	.	.	.	G	.	A	T	.
AK4	MH686538	C	T	C	A	T	C	C	T	.	.	G	.	.	.	A	.	G	G	.	G	.	A	.	.
DAG1	MH686539	C	T	C	A	T	C	.	T	C	.	G	.	T	G	A	G	.	.	.	G	.	.	.	.
DAG2	MH686540	C	T	C	A	T	C	.	T	C	.	G	.	T	G	A	G	.	.	.	G	G	.	.	.
DAG3	MH686541	C	T	C	A	T	C	.	T	C	.	G	C	T	G	A	.	.	.	.	G	G	.	.	.
AWA1	MH686542	C	T	C	A	T	C	.	T	C	.	G	.	.	G	A	.	G	G	A	G	G	A	.	.
AWA2	MH686543	C	T	C	A	T	C	.	T	.	.	G	.	T	G	A	.	G	G	A	G	G	.	.	.
AWA3	MH686544	C	T	C	A	T	C	.	T	.	.	G	.	T	G	A	.	G	G	A	G	G	A	.	T
SAK1	MH686545	C	T	C	A	T	C	.	T	C	.	G	.	.	.	A	.	.	.	.	G	.	A	.	.
SAK2	MH686546	C	T	C	A	T	C	.	T	C	.	G	.	T	G	A	.	.	.	A	G	.	A	.	.
SAK3	MH686547	C	T	C	A	T	C	.	T	.	.	G	.	.	.	A	.	.	G	A	G	.	A	.	.
KIV1	MH686548	C	T	C	A	T	C	.	T	C	.	G	.	T	G	A	G	.	G	A	G	.	.	.	.
KIV2	MH686549	C	T	C	A	T	C	.	T	C	.	G	.	T	G	A	G	.	G	A	G	.	.	.	T
KIV3	MH686550	C	T	C	A	T	C	.	T	C	.	G	.	T	G	A	G	.	.	A	G	.	.	T	T
KAR1	MH686551	C	T	C	A	T	C	.	T	.	.	G	.	T	G	.	.	.	G	A	G	.	.	T	.
KAR2	MH686552	C	T	C	A	T	C	.	T	.	.	G	.	.	.	.	.	.	.	.	G	G	.	T	.
KAR3	MH686553	C	T	C	A	T	C	.	T	.	.	G	.	.	.	A	.	.	.	.	G	G	.	T	.
MAL1	MH686554	C	T	C	A	T	C	.	T	.	.	G	.	T	G	.	.	.	.	.	G	.	A	.	.
MAL2	MH686555	C	T	C	A	T	C	.	T	.	.	G	.	.	.	.	.	.	.	.	G	.	.	.	.
MAL3	MH686556	C	T	C	A	T	C	.	.	.	.	G	.	.	.	A	.	.	.	.	G	.	.	.	.
MOR1	MH686557	C	T	C	A	T	C	.	T	.	.	G	.	.	G	A	.	.	G	A	G	.	A	.	T
MOR2	MH686558	C	T	C	A	T	C	.	T	.	.	G	C	.	.	A	.	.	.	.	G	.	A	.	.
MOR3	MH686559	C	T	C	A	T	C	.	T	C	.	G	.	.	.	A	.	.	.	.	G	G	.	T	T

${}^{*}$ Nucleotide positions are taken from the NCBI GenBank record AM884914 (Ballingall et al., 2008).

The major histocompatibility complex (MHC) consists of a group of closely linked genes, and these genes are highly polymorphic. The main function of the MHC is to code specialized antigen-presenting receptor glycoproteins, which are known as histocompatibility molecules or MHC molecules (Dukkipati et al., 2006). The products of MHC genes play a key role in the immune system of the individuals. MHC genes have been extensively studied as candidate genes for disease resistance in many animal breeds (Dongxiao and Yuan, 2004). DRB genes have been commonly used and characterized in the MHC region. The second exon of the *DRB* gene, in particular, has been the most widely studied gene region because it encodes peptide-binding sites that are extremely polymorphic (Blattman et al., 1993; Amills et al., 1996; Dongxiao and Yuan, 2004). The MHC of sheep is located on chromosome 20 and is abbreviated as *Ovar* or *OLA* genes (Brujeni et al., 2009).

*Ovar-DRB1* was found to be associated with gastrointestinal nematodes (Buitkamp et al., 1994; Schwaiger et al., 1994; Stear et al., 1996; Charon et al., 2002; Sayers et al., 2005) and BLV (bovine leukemia virus) induced ovine lymphoma (Nagaoka et al., 1999; Konnai et al., 2003a). Therefore, MHC loci have become attractive for studies of DNA markers for gastrointestinal nematodes and BLV-induced ovine lymphoma (Dukkipati et al., 2006).

**Figure 1:**
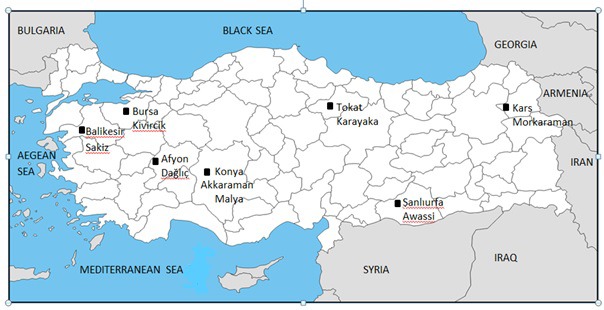
The location of the samples used for DNA sequencing (drawn by Fatma İhan).

**Figure 2:**
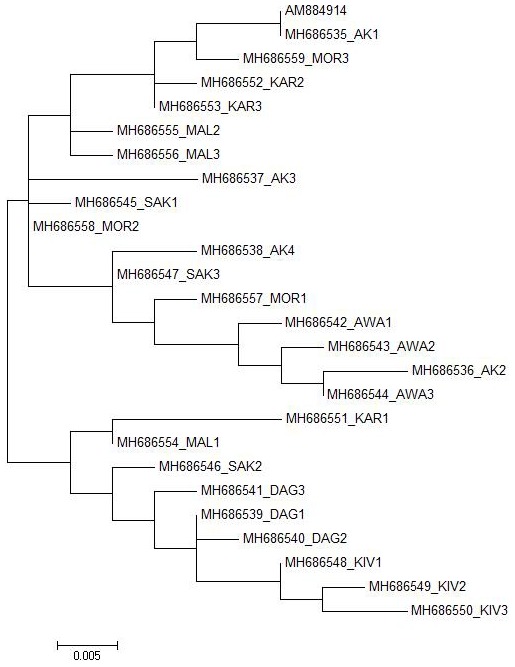
Maximum likelihood tree estimated under the HKY+I model for the *Ovar-DRB1* gene exon 2 sequences of Turkish native sheep breeds with the AM884914 GenBank record.

Genetic studies of variation in *Ovar-MHC* class II genes have shown that the *DRB* locus is highly polymorphic (Schwaiger et al., 1994; Schwaiger and Epplen, 1995; Amills et al., 1996; Jugo and Vicario, 2000; Konnai et al., 2003b; Ballingall et al., 2008; Nikbakht et al., 2012; Lotfi et al., 2012; Shen et al., 2014; Takeshima et al., 2014). In particular, most of the polymorphism is found in exon 2, which encodes the antigen-binding site (Escayg et al., 1997; Konnai et al., 2003b, c).

Many studies have been carried out on the exon 2 of the *DRB* locus in different sheep breeds from all over the world (Blattman et al., 1993; Amills et al., 1996; Konnai et al. 2003b, c; Dongxiao and Yuan, 2004; Gruszczyńska et al., 2005; Shen et al., 2014). Nevertheless, there is little information about the MHC *DRB* polymorphism on Turkish native sheep breeds. To date, only two studies have been found in Turkish native sheep breeds (Bozkaya and Kurar, 2005; Ilhan et al., 2016).

## 2 Material and methods

In this research, eight different native sheep breeds that are reared in Turkey, including the Akkaraman, Daǧliç, Awassi, Sakiz, Kivircik, Karayaka, Malya and Morkaraman (10 sheep per breed) breeds, were studied via DNA sequencing. The sampling locations and provinces are given on the map of Turkey (Fig. 1).

Total DNA was extracted from blood samples as described in Miller et al. (1988). The concentration and purification of genomic DNA was quantified with a NanoDrop ND-1000 spectrophotometer, and 20–30 ng of genomic DNA was used for the polymerase chain reaction (PCR). *Ovar-DRB1* was amplified according to Konnai et al. (2003b). We used nested PCR to amplify the second exon of the *DRB1* gene. The first PCR was performed with the primers OLA-ERB1 and HL031:

- OLA-ERB1 (5${}^{\prime }$-CCGGAATTCCCGTCTCTGCAGCAC ATTTCTT-3${}^{\prime }$)
- HL031 (5${}^{\prime }$-TTTAAATTCGCGCTCACCTCGCCGCT-3${}^{\prime }$).

All the PCRs were performed in 25 µL volume containing 2 µL of 10× PCR buffer with KCl, 1.5 mM MgCl${}_{2}$, 1.2 mM of dNTP mix, 20 µM of each primer, 2.5 U of Taq polymerase and 20 ng of DNA. The PCR amplification profile was initial denaturation for 5 min at 94 ${}^{\circ }$C, 94 ${}^{\circ }$C for 30 s (15 cycles), primer annealing at 50 ${}^{\circ }$C for 30 s and 1 min at 72 ${}^{\circ }$C for extension, and a final extension step at 72 ${}^{\circ }$C for 10 min.

The second round of PCR was carried out using 5 µL of the resultant mixture, with the addition of primers OLA-ERB1 and OLA-XRB1:

- OLA-ERB1 (5${}^{\prime }$-CCGGAATTCCCGTCTCTGCAGCAC ATTTCTT-3${}^{\prime }$)
- OLA-XRB1 (5${}^{\prime }$-GCTCGAGCGCTGCACAGTGAAAC TC-3${}^{\prime }$).

The thermal cycle profile started with an initial denaturation at 94 ${}^{\circ }$C for 5 min after 94 ${}^{\circ }$C for 30 s, annealing at 60 ${}^{\circ }$C for 30 s and extension at 72 ${}^{\circ }$C for 1 min (30 cycles), followed by a final extension step at 72 ${}^{\circ }$C for 10 min. The amplified PCR products were electrophoresed on 1 % agarose gels to verify the fragment sizes.

In this study, genetic variability in the ovine MHC, class II *DRB1* gene was analyzed by DNA sequencing. Amplicons were purified using the PCR cleanup kit and subjected to direct sequence analysis on an ABI Prism 3100 genetic analyzer (Applied Biosystems^™^, USA) using standard protocols in order to verify the sequence variations. Sequences were aligned with BioEdit Sequence Alignment Editor with Clustal W multiple alignment modules (Hall, 1999). The phylogenetic analysis was performed using MEGA 6 Software (Molecular Evolutionary Genetic Analysis, version 6.0) (Tamura et al., 2013) and the tree was constructed by the maximum likelihood (ML) approach on the basis of genetic distance matrix (Felsenstein, 1981).

## 3 Results

We analyzed the DNA sequence polymorphism of the *Ovar-DRB1* gene in the MHC gene region in a total of 80 animals from eight different native Turkish sheep breeds: Akkaraman (AK), Daǧliç (DAG), Awassi (AWA), Sakiz (SAK), Kivircik (KIV), Karayaka (KAR), Malya (MAL) and Morkaraman (MOR). The highly polymorphic exon 2 region, which includes antigen recognition sites (ARSs) in the MHC gene region, was used for sequence analysis. The *Ovar-DRB1* locus is 11.979 bp long. We used 286 bp (primers excluded) of the exon 2 region, which is widely used in molecular studies for comparing different ruminant breeds. A total of 25 different alleles were revealed for this segment in Turkish native sheep breeds with 24 variable sites (Table 1). Only 13 sites were parsimony informative. The average pairwise genetic distance was 0.029 % for the *Ovar-DRB1* gene exon 2 region. The DNA sequences of the alleles were deposited in the NCBI GenBank database, and DNA sequence variations and GenBank numbers are given in Table 1. The exon 2 of *Ovar-DRB1* gene was aligned, and the nucleotide positions were taken from Ballingall et al. (2008). The NCBI GenBank record AM884914 is taken for comparison of the nucleotide positions because the whole *DRB* gene is given in this GenBank record.

The sequence variations at eight different positions (7026, 7036, 7040, 7053, 7059, 7069, 7131 and 7214) are found in all of the studied samples. G → C transversion at position 7081 is only seen in the Akkaraman sheep breed, whereas T → C transition at position 7097 is only seen in one sample from the Akkaraman breed. The other variable sites are given in Table 1.

The best DNA/protein model was the maximum likelihood model for constructing the phylogenetic trees using the MEGA 6 software.

In total, 25 different alleles were identified in exon 2 of the *Ovar-DRB1* gene of Turkish native sheep breeds. The 25 alleles were mainly clustered in two branches in the ML tree (Fig. 2). All Daǧliç and Kivircik alleles and one allele from Karayaka, Malya and Sakiz were mainly clustered in one branch while all the other alleles were clustered in the other branch. The other breeds in the second branch did not diverge quite as strongly from each other, except for Awassi.

## 4 Discussion

The MHC gene family is a well-studied gene region in the vertebrate immune system and encodes antigen recognition proteins used in the adaptive immune response. Polymorphism studies of this gene have become very popular in the past decades. A variety of studies have shown that the MHC of sheep and goats introduced much more mutation and polymorphism than other genes.

In two of the recent studies of Turkish native sheep breeds, the restriction fragment length polymorphism (RFLP) pattern of the MHC gene region in the *DRB1* and *DRB3* loci in Turkish sheep populations (Ilhan et al., 2016) and the presence of linkage disequilibrium between nine microsatellite loci in and outside the MHC gene region (Bozkaya and Kurar, 2005) were shown in Akkaraman, Awassi and Merinolandschaf sheep populations.

In the present study, we have used DNA sequencing of the *Ovar-DRB1* gene exon 2 region to verify the genetic variation within and between some of the Turkish native sheep breeds. Overall, two main groups were detected among the 25 alleles from Turkish native sheep breeds (Fig. 2). All Daǧliç and Kivircik alleles and one allele from Karayaka, Malya and Sakiz are grouped together while all the other breeds are grouped in the other branches.

In our study a total of 80 sheep from eight breeds of Turkey were analyzed and 25 new alleles were identified. These alleles were 98 to 96.1 % identical at the nucleotide level in the GenBank records. The sequence variations at eight different positions (7026, 7036, 7040, 7053, 7059, 7069, 7131 and 7214) are found in all of the studied samples of Turkish native sheep breeds. These variable sites are found as fixed alleles in Turkish native sheep breeds.

In addition to previous findings on the exon 2 of *Ovar-DRB1* gene, we report here a sequence analysis of this region in Turkish native sheep breeds and compare the results with published records. Our data show that two main groups were detected in Turkish native sheep breeds. Therefore, it is highly important to identify the genetic structure of Turkish native sheep breeds and improve strategies to conserve them in their local areas. Thus, we contribute to the National Sheep and Goat Breeding Project of Turkey with this study.

## Supplement

10.5194/aab-61-475-2018-supplement The supplement related to this article is available online at: https://doi.org/10.5194/aab-61-475-2018-supplement.

## Data Availability

In the file the photo of the PCR product, Bioedit Sequence Alignment results of some of the samples and the sequence results of the studied samples are given in the Supplement.
