# Supplementary material for: Genetic characterization of some Turkish sheep breeds based on the sequencing of the Ovar-DRB1 gene in the major histocompatibility complex (MHC) gene region
Source: Arch Anim Breed. 2018 Dec 6;61(4):475–80. doi: 10.5194/aab-61-475-2018 (PMC7065386; doi:10.5194/aab-61-475-2018)
Supplement: The supplement related to this article is available online at: https://doi.org/10.5194/aab-61-475-2018-supplement. [file aab-61-475-supplement.zip › aab-61-475-2018-supplement-title-page.pdf]

Supplement of Arch. Anim. Breed., 61, 475–480, 2018
https://doi.org/10.5194/aab-61-475-2018-supplement
© Author(s) 2018. This work is distributed under
the Creative Commons Attribution 4.0 License.

Open Access
Archives Animal Breeding

Supplement of

Genetic characterization of some Turkish sheep breeds based on the sequencing of the *Ovar-DRB1* gene in the major histocompatibility complex (MHC) gene region

Fulya Özdil et al.

Correspondence to: Fulya Özdil (fozdil@nku.edu.tr)

- aab-61-475-2018-supplement-title-page.pdf
- Supplement file.docx

The copyright of individual parts of the supplement might differ from the CC BY 4.0 License.
