# Supplementary material for: Genetic characterization of some Turkish sheep breeds based on the sequencing of the Ovar-DRB1 gene in the major histocompatibility complex (MHC) gene region
Source: Arch Anim Breed. 2018 Dec 6;61(4):475–80. doi: 10.5194/aab-61-475-2018 (PMC7065386; doi:10.5194/aab-61-475-2018)
Supplement: The supplement related to this article is available online at: https://doi.org/10.5194/aab-61-475-2018-supplement. [file aab-61-475-supplement.zip › Supplement file.docx]

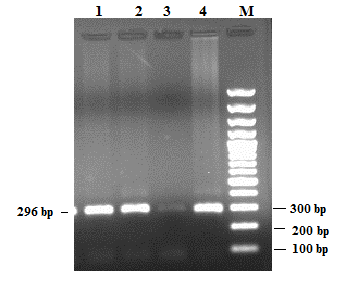


**Fig. 1.** PCR Product of *Ovar-DRB*1 gene exon 2 region


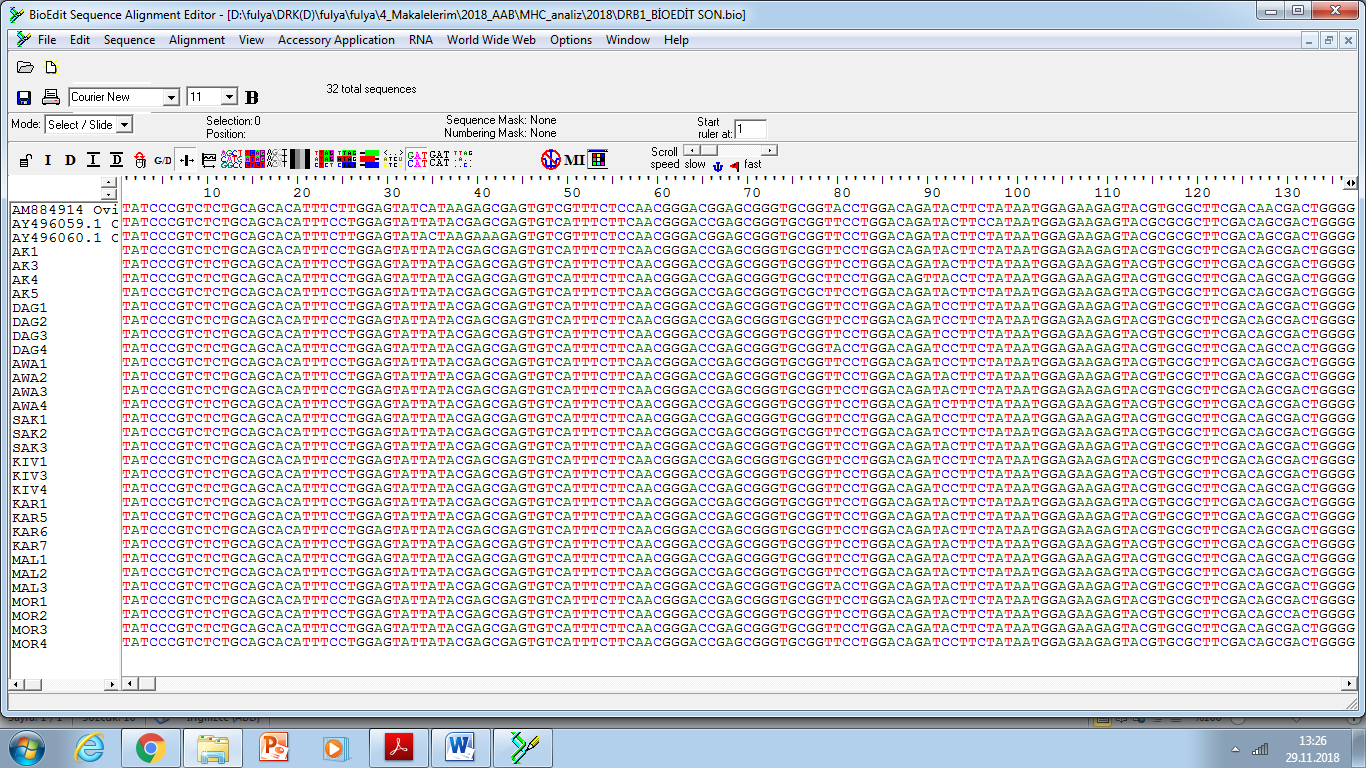


**Fig 2.** Bioedit Sequence Alignment results of the samples.

**Fig. 3**. The sequence results of the studied samples are given below.

>MH686535 AK1

TATCCCGTCTCTGCAGCACATTTCCTGGAGTATTATACGAGCGAGTGTCATTTCTTCAAC

GGGACCGAGCGGGTGCGGTTCCTGGACAGATACTTCTATAATGGAGAAGAGTACGTGCGC

TTCGACAGCGACTGGGGCGAGTACCGAGCGGTGGCCGAGCTGGGGCGGCCGGACGCCAAG

TACTGGAACAGCCAGAAGGACTTCCTGGAGCGGACGCGGGCCGATGTGGACACGTACTGC

AGACACAACTACGGGGTCTTTGAGAGTTTCAGTGTGCAGCGGCGAA

>MH686536 AK2

TATCCCGTCTCTGCAGCACATTTCCTGGAGTATTATACGAGCGAGTGTCATTTCTTCAAC

GGGACCGAGCGGGTGCGGTTCCTGGACAGATACTTCTATAATGGAGAAGAGTACGTGCGC

TTCGACAGCGACTGGGGCGAGTTCCGGGCGGTGGCCGAGCTGGGGCGGCCGAACGCCGAG

TACTGGAACAGCCAGAAGGAGATCCTGGAGCGGACGCGGACCGATGTGGACACGTACTGC

AGACACAACTACGGGGTCTTTGAGAGTTTCAGTGTGCAGCGGCGAA

>MH686537 AK3

TATCCCGTCTCTGCAGCACATTTCCTGGAGTATTATACGAGCGAGTGTCATTTCTTCAAC

GGGACCGAGCGGGTGCGCTTCCTGGACAGTTACCTCTATAATGGAGAAGAGTACGTGCGC

TTCGACAGCGACTGGGGCGAGTACCGAGCGGTGGCCGAGCTGGGGCGGCCGAACGCCAAG

TACTGGAACAGCCAGAAGGACTTCCTGGAGCGGACGCGGACCGATGTGGACACGTACTGC

AGACACAACTACGGGGTCATTGAGAGTTTCAGTGTGCAGCGGCGAA

>MH686538 AK4

TATCCCGTCTCTGCAGCACATTTCCTGGAGTATTATACGAGCGAGTGTCATTTCTTCAAC

GGGACCGAGCGGGTGCGCTTCCTGGACAGATACTTCTATAATGGAGAAGAGTACGTGCGC

TTCGACAGCGACTGGGGCGAGTACCGAGCGGTGGCCGAGCTGGGGCGGCCGAACGCCGAG

TACTGGAACAGCCAGAAGGAGATCCTGGAGCGGACGCGGACCGAGGTGGACACGTACTGC

AGACACAACTACGGGGTCATTGAGAGTTTCAGTGTGCAGCGGCGAA

>MH686539 DAG1

TATCCCGTCTCTGCAGCACATTTCCTGGAGTATTATACGAGCGAGTGTCATTTCTTCAAC

GGGACCGAGCGGGTGCGGTTCCTGGACAGATCCTTCTATAATGGAGAAGAGTACGTGCGC

TTCGACAGCGACTGGGGCGAGTTCCGGGCGGTGGCCGAGCTGGGGCGGCCGAGCGCCAAG

TACTGGAACAGCCAGAAGGACTTCCTGGAGCGGACGCGGGCCGAGGTGGACACGTACTGC

AGACACAACTACGGGGTCATTGAGAGTTTCAGTGTGCAGCGGCGAA

>MH686540 DAG2

TATCCCGTCTCTGCAGCACATTTCCTGGAGTATTATACGAGCGAGTGTCATTTCTTCAAC

GGGACCGAGCGGGTGCGGTTCCTGGACAGATCCTTCTATAATGGAGAAGAGTACGTGCGC

TTCGACAGCGACTGGGGCGAGTTCCGGGCGGTGGCCGAGCTGGGGCGGCCGAGCGCCAAG

TACTGGAACAGCCAGAAGGACTTCCTGGAGCGGAGGCGGGCCGAGGTGGACACGTACTGC

AGACACAACTACGGGGTCATTGAGAGTTTCAGTGTGCAGCGGCGAA

>MH686541 DAG3

TATCCCGTCTCTGCAGCACATTTCCTGGAGTATTATACGAGCGAGTGTCATTTCTTCAAC

GGGACCGAGCGGGTGCGGTTCCTGGACAGATCCTTCTATAATGGAGAAGAGTACGTGCGC

TTCGACAGCGACTGGGGCGAGTTCCGGGCGGTGGCCGAGCTGGGGCGGCCGAACGCCAAG

TACTGGAACAGCCAGAAGGACTTCCTGGAGCGGAGGCGGGCCGAGGTGGACACGTACTGC

AGACACAACTACGGGGTCATTGAGAGTTTCAGTGTGCAGCGGCGAA

>MH686542 AWA1

TATCCCGTCTCTGCAGCACATTTCCTGGAGTATTATACGAGCGAGTGTCATTTCTTCAAC

GGGACCGAGCGGGTGCGGTTCCTGGACAGATCCTTCTATAATGGAGAAGAGTACGTGCGC

TTCGACAGCGACTGGGGCGAGTACCGGGCGGTGGCCGAGCTGGGGCGGCCGAACGCCGAG

TACTGGAACAGCCAGAAGGAGATCCTGGAGCGGAGGCGGACCGAGGTGGACACGTACTGC

AGACACAACTACGGGGTCATTGAGAGTTTCAGTGTGCAGCGGCGAA

>MH686543 AWA2

TATCCCGTCTCTGCAGCACATTTCCTGGAGTATTATACGAGCGAGTGTCATTTCTTCAAC

GGGACCGAGCGGGTGCGGTTCCTGGACAGATACTTCTATAATGGAGAAGAGTACGTGCGC

TTCGACAGCGACTGGGGCGAGTTCCGGGCGGTGGCCGAGCTGGGGCGGCCGAACGCCGAG

TACTGGAACAGCCAGAAGGAGATCCTGGAGCGGAGGCGGGCCGAGGTGGACACGTACTGC

AGACACAACTACGGGGTCATTGAGAGTTTCAGTGTGCAGCGGCGAA

>MH686544 AWA3

TATCCCGTCTCTGCAGCACATTTCCTGGAGTATTATACGAGCGAGTGTCATTTCTTCAAC

GGGACCGAGCGGGTGCGGTTCCTGGACAGATACTTCTATAATGGAGAAGAGTACGTGCGC

TTCGACAGCGACTGGGGCGAGTTCCGGGCGGTGGCCGAGCTGGGGCGGCCGAACGCCGAG

TACTGGAACAGCCAGAAGGAGATCCTGGAGCGGAGGCGGACCGAGGTGGACACGTACTGC

AGACACAACTACGGGGTCTTTGAGAGTTTCAGTGTGCAGCGGCGAA

>MH686545 SAK1

TATCCCGTCTCTGCAGCACATTTCCTGGAGTATTATACGAGCGAGTGTCATTTCTTCAAC

GGGACCGAGCGGGTGCGGTTCCTGGACAGATCCTTCTATAATGGAGAAGAGTACGTGCGC

TTCGACAGCGACTGGGGCGAGTACCGAGCGGTGGCCGAGCTGGGGCGGCCGAACGCCAAG

TACTGGAACAGCCAGAAGGACTTCCTGGAGCGGACGCGGACCGAGGTGGACACGTACTGC

AGACACAACTACGGGGTCATTGAGAGTTTCAGTGTGCAGCGGCGAA

>MH686546 SAK2

TATCCCGTCTCTGCAGCACATTTCCTGGAGTATTATACGAGCGAGTGTCATTTCTTCAAC

GGGACCGAGCGGGTGCGGTTCCTGGACAGATCCTTCTATAATGGAGAAGAGTACGTGCGC

TTCGACAGCGACTGGGGCGAGTTCCGGGCGGTGGCCGAGCTGGGGCGGCCGAACGCCAAG

TACTGGAACAGCCAGAAGGACATCCTGGAGCGGACGCGGACCGAGGTGGACACGTACTGC

AGACACAACTACGGGGTCATTGAGAGTTTCAGTGTGCAGCGGCGAA

>MH686547 SAK3

TATCCCGTCTCTGCAGCACATTTCCTGGAGTATTATACGAGCGAGTGTCATTTCTTCAAC

GGGACCGAGCGGGTGCGGTTCCTGGACAGATACTTCTATAATGGAGAAGAGTACGTGCGC

TTCGACAGCGACTGGGGCGAGTACCGAGCGGTGGCCGAGCTGGGGCGGCCGAACGCCAAG

TACTGGAACAGCCAGAAGGAGATCCTGGAGCGGACGCGGACCGAGGTGGACACGTACTGC

AGACACAACTACGGGGTCATTGAGAGTTTCAGTGTGCAGCGGCGAA

>MH686548 KIV1

TATCCCGTCTCTGCAGCACATTTCCTGGAGTATTATACGAGCGAGTGTCATTTCTTCAAC

GGGACCGAGCGGGTGCGGTTCCTGGACAGATCCTTCTATAATGGAGAAGAGTACGTGCGC

TTCGACAGCGACTGGGGCGAGTTCCGGGCGGTGGCCGAGCTGGGGCGGCCGAGCGCCAAG

TACTGGAACAGCCAGAAGGAGATCCTGGAGCGGACGCGGGCCGAGGTGGACACGTACTGC

AGACACAACTACGGGGTCATTGAGAGTTTCAGTGTGCAGCGGCGAA

>MH686549 KIV2

TATCCCGTCTCTGCAGCACATTTCCTGGAGTATTATACGAGCGAGTGTCATTTCTTCAAC

GGGACCGAGCGGGTGCGGTTCCTGGACAGATCCTTCTATAATGGAGAAGAGTACGTGCGC

TTCGACAGCGACTGGGGCGAGTTCCGGGCGGTGGCCGAGCTGGGGCGGCCGAGCGCCAAG

TACTGGAACAGCCAGAAGGAGATCCTGGAGCGGACGCGGGCCGAGGTGGACACGTACTGC

AGACACAACTACGGGGTCTTTGAGAGTTTCAGTGTGCAGCGGCGAA

>MH686550 KIV3

TATCCCGTCTCTGCAGCACATTTCCTGGAGTATTATACGAGCGAGTGTCATTTCTTCAAC

GGGACCGAGCGGGTGCGGTTCCTGGACAGATCCTTCTATAATGGAGAAGAGTACGTGCGC

TTCGACAGCGACTGGGGCGAGTTCCGGGCGGTGGCCGAGCTGGGGCGGCCGAGCGCCAAG

TACTGGAACAGCCAGAAGGACATCCTGGAGCGGACGCGGGCCGATGTGGACACGTACTGC

AGACACAACTACGGGGTCTTTGAGAGTTTCAGTGTGCAGCGGCGAA

>MH686551 KAR1

TATCCCGTCTCTGCAGCACATTTCCTGGAGTATTATACGAGCGAGTGTCATTTCTTCAAC

GGGACCGAGCGGGTGCGGTTCCTGGACAGATACTTCTATAATGGAGAAGAGTACGTGCGC

TTCGACAGCGACTGGGGCGAGTTCCGGGCGGTGGCCGAGCTGGGGCGGCCGGACGCCAAG

TACTGGAACAGCCAGAAGGAGATCCTGGAGCGGACGCGGGCCGATGTGGACACGTACTGC

AGACACAACTACGGGGTCATTGAGAGTTTCAGTGTGCAGCGGCGAA

>MH686552 KAR2

TATCCCGTCTCTGCAGCACATTTCCTGGAGTATTATACGAGCGAGTGTCATTTCTTCAAC

GGGACCGAGCGGGTGCGGTTCCTGGACAGATACTTCTATAATGGAGAAGAGTACGTGCGC

TTCGACAGCGACTGGGGCGAGTACCGAGCGGTGGCCGAGCTGGGGCGGCCGGACGCCAAG

TACTGGAACAGCCAGAAGGACTTCCTGGAGCGGAGGCGGGCCGATGTGGACACGTACTGC

AGACACAACTACGGGGTCATTGAGAGTTTCAGTGTGCAGCGGCGAA

>MH686553 KAR3

TATCCCGTCTCTGCAGCACATTTCCTGGAGTATTATACGAGCGAGTGTCATTTCTTCAAC

GGGACCGAGCGGGTGCGGTTCCTGGACAGATACTTCTATAATGGAGAAGAGTACGTGCGC

TTCGACAGCGACTGGGGCGAGTACCGAGCGGTGGCCGAGCTGGGGCGGCCGAACGCCAAG

TACTGGAACAGCCAGAAGGACTTCCTGGAGCGGAGGCGGGCCGATGTGGACACGTACTGC

AGACACAACTACGGGGTCATTGAGAGTTTCAGTGTGCAGCGGCGAA

>MH686554 MAL1

TATCCCGTCTCTGCAGCACATTTCCTGGAGTATTATACGAGCGAGTGTCATTTCTTCAAC

GGGACCGAGCGGGTGCGGTTCCTGGACAGATACTTCTATAATGGAGAAGAGTACGTGCGC

TTCGACAGCGACTGGGGCGAGTTCCGGGCGGTGGCCGAGCTGGGGCGGCCGGACGCCAAG

TACTGGAACAGCCAGAAGGACTTCCTGGAGCGGACGCGGACCGAGGTGGACACGTACTGC

AGACACAACTACGGGGTCATTGAGAGTTTCAGTGTGCAGCGGCGAA

>MH686555 MAL2

TATCCCGTCTCTGCAGCACATTTCCTGGAGTATTATACGAGCGAGTGTCATTTCTTCAAC

GGGACCGAGCGGGTGCGGTTCCTGGACAGATACTTCTATAATGGAGAAGAGTACGTGCGC

TTCGACAGCGACTGGGGCGAGTACCGAGCGGTGGCCGAGCTGGGGCGGCCGGACGCCAAG

TACTGGAACAGCCAGAAGGACTTCCTGGAGCGGACGCGGGCCGAGGTGGACACGTACTGC

AGACACAACTACGGGGTCATTGAGAGTTTCAGTGTGCAGCGGCGAA

>MH686556 MAL3

TATCCCGTCTCTGCAGCACATTTCCTGGAGTATTATACGAGCGAGTGTCATTTCTTCAAC

GGGACCGAGCGGGTGCGGTACCTGGACAGATACTTCTATAATGGAGAAGAGTACGTGCGC

TTCGACAGCGACTGGGGCGAGTACCGAGCGGTGGCCGAGCTGGGGCGGCCGAACGCCAAG

TACTGGAACAGCCAGAAGGACTTCCTGGAGCGGACGCGGGCCGAGGTGGACACGTACTGC

AGACACAACTACGGGGTCATTGAGAGTTTCAGTGTGCAGCGGCGAA

>MH686557 MOR1

TATCCCGTCTCTGCAGCACATTTCCTGGAGTATTATACGAGCGAGTGTCATTTCTTCAAC

GGGACCGAGCGGGTGCGGTTCCTGGACAGATACTTCTATAATGGAGAAGAGTACGTGCGC

TTCGACAGCGACTGGGGCGAGTACCGGGCGGTGGCCGAGCTGGGGCGGCCGAACGCCAAG

TACTGGAACAGCCAGAAGGAGATCCTGGAGCGGACGCGGACCGAGGTGGACACGTACTGC

AGACACAACTACGGGGTCTTTGAGAGTTTCAGTGTGCAGCGGCGAA

>MH686558 MOR2

TATCCCGTCTCTGCAGCACATTTCCTGGAGTATTATACGAGCGAGTGTCATTTCTTCAAC

GGGACCGAGCGGGTGCGGTTCCTGGACAGATACTTCTATAATGGAGAAGAGTACGTGCGC

TTCGACAGCGACTGGGGCGAGTACCGAGCGGTGGCCGAGCTGGGGCGGCCGAACGCCAAG

TACTGGAACAGCCAGAAGGACTTCCTGGAGCGGACGCGGACCGAGGTGGACACGTACTGC

AGACACAACTACGGGGTCATTGAGAGTTTCAGTGTGCAGCGGCGAA

>MH686559 MOR3

TATCCCGTCTCTGCAGCACATTTCCTGGAGTATTATACGAGCGAGTGTCATTTCTTCAAC

GGGACCGAGCGGGTGCGGTTCCTGGACAGATCCTTCTATAATGGAGAAGAGTACGTGCGC

TTCGACAGCGACTGGGGCGAGTACCGAGCGGTGGCCGAGCTGGGGCGGCCGAACGCCAAG

TACTGGAACAGCCAGAAGGACTTCCTGGAGCGGAGGCGGGCCGATGTGGACACGTACTGC

AGACACAACTACGGGGTCTTTGAGAGTTTCAGTGTGCAGCGGCGAA
